# Ameliorative Effects of *Pueraria lobata* Extract on Postmenopausal Symptoms through Promoting Estrogenic Activity and Bone Markers in Ovariectomized Rats

**DOI:** 10.1155/2021/7924400

**Published:** 2021-09-06

**Authors:** Mi-Ra Lee, Bohye Kim, Yongjun Lee, Soung-Yong Park, Jae-Hoon Shim, Bong-Hwan Chung, Seong-Il Heo

**Affiliations:** ^1^Hongcheon Institute of Medicinal Herb, Hongcheon-gun 25142, Gangwon, Republic of Korea; ^2^Sejun F & B Co. Ltd., Hongcheon-gun 25107, Gangwon, Republic of Korea; ^3^Department of Food Science and Nutrition, Hallym University, Chuncheon 24252, Gangwon, Republic of Korea

## Abstract

*Pueraria lobata* (Willd.) Ohwi, known as *kudzu*, is one of the most popular traditional medicines in Asian countries. It has been widely used as a natural alternative to hormone replacement therapy for treating postmenopausal symptoms. This study aimed to investigate the estrogenic effect of *P. lobata* extract (PE) against postmenopausal osteoporosis in ovariectomized (OVX) rats. OVX rats were treated with PE (25–1600 mg/kg) for 8 weeks. Biochemical parameters, estradiol, and bone turnover markers (e.g., osteocalcin, C-terminal telopeptide fragment of type I collagen, deoxypyridinoline, and pyridinoline) were measured in plasma samples. In addition, estrogen receptor-alpha (ER-*α*) protein expression and morphology of uterine were evaluated. Long-term treatment with PE did not cause liver damage in OVX rats. PE supplementation reduced body weight gain in obese rats with high lipid accumulation induced by ovariectomy. Furthermore, PE exhibited a protective effect against insulin resistance, hyperlipidemia, and hepatic lipid peroxidation. PE treatment increased uterine weight and thickness of the uterine layers in cases of uterus atrophy due to removal of ovaries. The levels of bone turnover markers, which were significantly increased in OVX rats, were decreased by PE treatment. Western blotting analysis showed that ER-*α* protein expression was upregulated in PE-treated rats compared with OVX rats. These results suggest that PE could be a promising alternative functional food for improving menopausal symptoms.

## 1. Introduction

Menopause, also known as climacteric, is a biological stage that represents the end of the reproductive lifespan in women [1]. Menopause-induced estrogen deficiency may increase the risk of metabolic diseases, such as obesity, osteoporosis, cardiovascular disease, and diabetes [2, 3]. Hormone replacement therapy (HRT) is commonly used to treat menopausal symptoms, but long-term HRT can lead to an increased risk of carcinogenesis and cardiovascular diseases [4, 5]. Thus, there is a need for natural alternative therapies, including dietary supplements and new herbal medicines, to reduce adverse side effects. Phytoestrogens are estrogen-like compounds derived from plants with a structure similar to that of 17*β*-estradiol [6]. The antipostmenopausal effects of phytoestrogens have been demonstrated to have various biological activities, such as bone-protective effects and improvement in menopausal symptoms, as a potential alternative to HRT [7–9].

*Pueraria lobata* (Willd.) Ohwi, known as *kudzu*, has been traditionally used in combination with other herbs to produce synergistic and additive effects through a multitarget approach for disease [10]. It contains high levels of several isoflavones, such as puerarin, daidzin, daidzein, genistin, and genistein [11]. *Pueraria lobata* is known to have multiple pharmacological activities that help alleviate various diseases such as liver diseases, cardiovascular diseases, diabetes, and osteoporosis [12, 13]. In particular, puerarin, the primary active component of *P. lobata,* has been demonstrated to have preventive activity against osteoporosis in several studies using ovariectomized (OVX) animal models [14, 15]. An OVX rat model is considered appropriate for investigating problems related to postmenopausal bone loss, a primary risk factor for osteoporosis [16]. However, there is little information on the effect of *P. lobata* on dyslipidemia and bone metabolism in OVX rats. Based on the estrogenic activity of puerarin, we predicted that *P. lobata* extract (PE) could have beneficial effects on menopausal metabolic dysfunction, the endocrine system, and osteoporosis in OVX rats. Therefore, we investigated the protective effects of long-term supplementation with PE against metabolic parameters in OVX rats. In addition, white adipose tissue weight, atrophic uterus morphology, bone turnover markers, and estrogen levels were evaluated to clarify the underlying mechanisms of action of PE extract.

## 2. Materials and Methods

### 2.1. Preparation of *P. lobata* Extract

PE was provided by Sejun F & B Co., Ltd. (Gangwon, Korea). It was in the form of a spray-dried powder, which was prepared as follows. To separate the starch, dried *P. lobate* was soaked in 50% ethanol for 1 h and then centrifuged. The residue of *P. lobate* was extracted in five volumes of 50% ethanol for 1 h at 60°C. The extract was filtered and concentrated under reduced pressure. The PE was powdered in the spray drying system by mixing 30% dextrin. The content of puerarin, which is a major isoflavone in PE, was 42.38 mg/g.

### 2.2. Animals and Treatment

Nine-week-old female Sprague Dawley rats, which were ovariectomized or sham-operated at 8 weeks of age, were purchased from Chemon Inc. (Gyeonggi-do, Korea) and maintained under controlled laboratory conditions (23°C ± 1°C, humidity 55% ± 5%, 12 h light/dark cycle) for 2 weeks before the experiments. The rats were randomly divided into the following treatment groups (*n* = 8 per group): (1) sham group, (2) OVX group, (3) OVX + PE 25 mg/kg group, (4) OVX + PE 100 mg/kg group, (5) OVX + PE 400 mg/kg group, and (6) OVX + PE 1600 mg/kg group. All animal experiment protocols in this study were reviewed and approved by the Institutional Animal Care and Use Committee of the Hongcheon Institute of Medicinal Herb (No. 19–07). PE was suspended in phosphate buffered saline (PBS) for oral administration at the desired dose in a volume of 5 mL/kg once daily for 8 weeks. The food intake and body weight of rats were measured twice a week. After 16 h of fasting on the last day of the experiment, blood was collected from the portal vein. The uterus and white adipose tissue were collected and weighed.

### 2.3. Biochemical Parameters

The plasma samples were prepared by centrifuging the collected blood in heparinized tubes (4,000 rpm for 15 min at 4°C) and then stored at −80°C for assay. Aspartate aminotransferase (AST), alanine aminotransferase (ALT), Ca, P, total cholesterol (TC), high-density lipoprotein cholesterol (HDL-C), low-density lipoprotein cholesterol (LDL-C), and triglyceride (TG) levels were measured using an automated clinical chemistry analyser (Konelab 20XT; Thermo Fisher Scientific, Waltham, MA, USA). Free fatty acid (FFA; BioAssay Systems, Hayward, CA, USA), insulin, estradiol (E2), and serotonin levels were determined using enzyme-linked immunosorbent assay (ELISA) kits (Cusabio, Wuhan, China) according to the manufacturer's instructions.

### 2.4. Liver Lipid Peroxidation Assay

The liver sample (120 mg) was homogenized in 1 mL of PBS and then centrifuged at 10,000 rpm for 15 min at 4°C. The supernatant obtained was used a source for the assay. Malondialdehyde (MDA) level was determined using the thiobarbituric acid reactive substance (TBARS) assay according to the manufacturer's (Dogenbio, Seoul, Korea) instructions.

### 2.5. Bone Turnover Markers in Plasma Samples

The bone formation marker Osteocalcin (OC) was determined using a rat osteocalcin ELISA kit (Cusabio). The bone resorption markers, such as C-terminal telopeptide fragment of type I collagen (CTX-1; Cusabio), deoxypyridinoline (DPD; Cusabio), and pyridinoline (PYD; MyBioSource, San Diego, USA), were determined using commercial ELISA kits.

### 2.6. Immunoblot Analysis

The total protein in the uterus was extracted using RIPA lysis buffer containing phosphatase and protease inhibitor cocktails. Equal amounts of protein (15 *μ*g) were separated on 10% sodium dodecyl sulfate polyacrylamide gels and then transferred on to polyvinylidene fluoride membranes. The membranes were blocked with 5% skim milk for 1 h and then probed with specific primary antibodies at a final dilution of 1 : 1000. The immunoreactive protein bands were detected using an enhanced chemiluminescence western blot detection kit (Clarity Max^TM^ Western ECL Substrate; Bio-Rad Laboratories, Hercules, CA, USA) and visualized using the Chemidoc Touch image system (ChemiDoc^TM^ XRS + system; Bio-Rad Laboratories).

### 2.7. Histological Analysis

The uterus samples were fixed in 10% formalin and embedded in paraffin. The tissue sections were mounted on glass slides and stained with haematoxylin and eosin. The static parameters of the uterus, endometrial, myometrial, and epithelial lengths were calculated using ImageJ software (NIH, USA).

### 2.8. Statistical Analysis

All data are expressed as mean ± standard error of the mean (SEM). They were analysed using the analysis of variance followed by Dunnett's multiple comparison test. Differences were considered statistically significant at *p* < 0.05. All statistical analyses were performed using GraphPad Prism 7.05.

## 3. Results

### 3.1. Effects of PE on Body Weight, Food Efficiency Ratio, and White Adipose Tissue in OVX Rats

**Figure 1:**
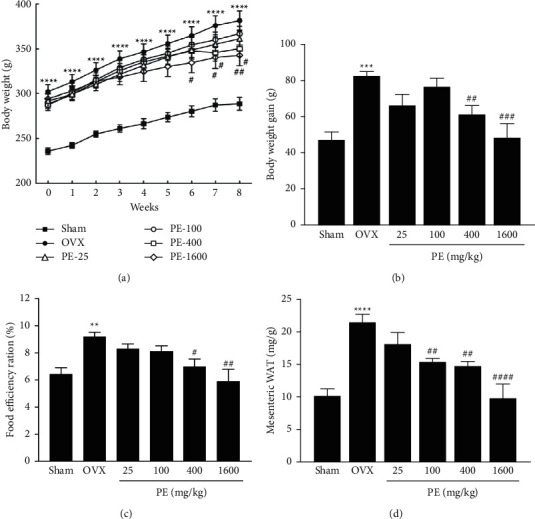
Effects of PE on (a) body weight changes, (b) body weight gain, (c) food efficiency ratio, and (d) abdominal white adipose tissue weight in OVX rats. Data represent the mean ± standard error of mean (SEM). ^*∗∗*^*p* < 0.01, ^*∗∗∗*^*p* < 0.001, and ^*∗∗∗∗*^*p* < 0.0001*vs*. sham group. ^#^*p* < 0.05, ^##^*p* < 0.01, ^###^*p* < 0.001, and ^####^*p* < 0.0001*vs*. OVX group.

As shown in Figure 1(a), the OVX group showed a significant increase in body weight compared with the sham group throughout the experimental period. However, PE treatment (400 and 1600 mg/kg) significantly reduced body weight gain from week 6 by 15% and 35%, respectively, compared with the OVX group (Figure 1(b)). The food efficiency ratio in the PE-1600 group was 6.28% ± 0.88%, which was significantly reduced by 28% compared to that in the OVX group (Figure 1(c)). The weight of mesenteric white adipose tissue (mWAT) increased significantly in the OVX group compared with the sham group. PE treatment significantly suppressed the increase in mWAT weight in the OVX rats (Figure 1(d)).

### 3.2. Effects of PE on Uterus Weight and Histological Analysis

**Figure 2:**
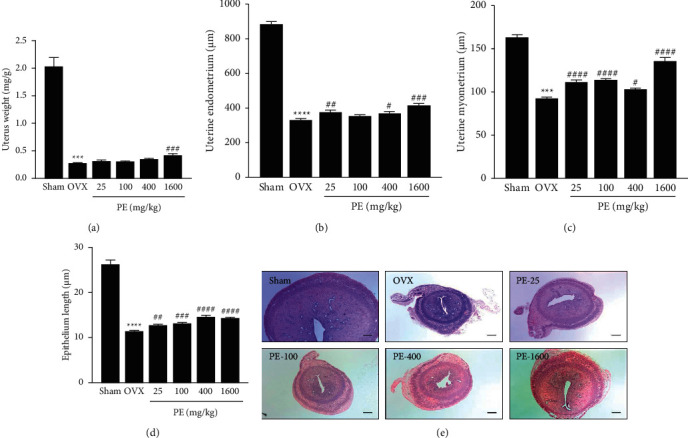
Effects of PE on uterus weight and histological analysis in OVX rats. (a) Uterus weight, (b) uterine endometrial, (c) uterine myometrial, (d) uterine epithelial length, and (e) haematoxylin and eosin stain (50×, bar = 200 *μ*m). Each column represents the mean ± standard error of mean (SEM). ^*∗∗∗*^*p* < 0.001 and ^*∗∗∗∗*^*p* < 0.0001*vs*. sham group; ^##^*p* < 0.01, ^###^*p* < 0.001, and ^####^*p* < 0.0001*vs*. OVX group.

The OVX rats presented with prominent atrophy of the uterus as shown in Figure 2(e). The uterus weight of rats in the OVX group (0.27 ± 0.22 mg/g) was 86% lower than that in the sham group (2.03 ± 0.49 mg/g) (Figure 2(a)). Although not statistically significant, PE treatment recovered the decrease in uterus weight by 9%–46% in a dose-dependent manner compared with the OVX group. Figures 2(b)–2(d) show the results of histological analysis of the stained uterine sections. The thickness of the endometrium, myometrium, and epithelium in the OVX group was significantly decreased compared with that in the sham group. However, PE treatment showed a marked reversal of the OVX-induced uterine atrophy.

### 3.3. Effects of PE on Plasma Biochemical Parameters

The results of plasma biochemical parameters are summarised in Table 1. The plasma levels of ALT and AST were not significantly different among the groups. The blood Ca level in the OVX group decreased by 10% compared with that in the sham group, but it was not statistically significant. Treatment with either PE 400 or 1600 mg/kg significantly increased the blood Ca level compared with the OVX group. The blood P level did not differ significantly among the groups. The OVX group showed a considerable increase in fasting plasma FFA and insulin levels compared with the sham group. The PE-1600 group showed a significant decrease in FFA level by 50% compared with the OVX group. The elevated insulin level in OVX rats was significantly reduced in all PE-treated groups except for the PE-100 group.

### 3.4. Effects of PE on Plasma Lipids and Liver MDA Levels in OVX Rats

To further investigate whether PE treatment improves the high lipid accumulation induced by ovariectomy, we measured the changes in plasma lipid profiles and MDA levels in the liver homogenates (Table 2). The plasma TC and LDL-C levels did not change in the OVX- and PE-treated groups. However, chronic PE treatment increased the HDL-C levels, with the PE-400 group, especially showing a statistically significant increase compared with the OVX group. The OVX group showed significantly elevated TG levels compared with the sham group. PE-treated groups inhibited increase of TG levels by 16% to 43%. Furthermore, the PE-25 group significantly reduced TG level to the similar results of the sham group (*p* < 0.001). Liver MDA levels significantly increased by 25% in the OVX group compared with the sham group. Treatment with PE significantly reduced these increases in MDA levels to values comparable to those in the sham group.

### 3.5. Effects of PE on Bone Mineral Density, OC, E2, and ER-*α* Protein Levels

**Figure 3:**
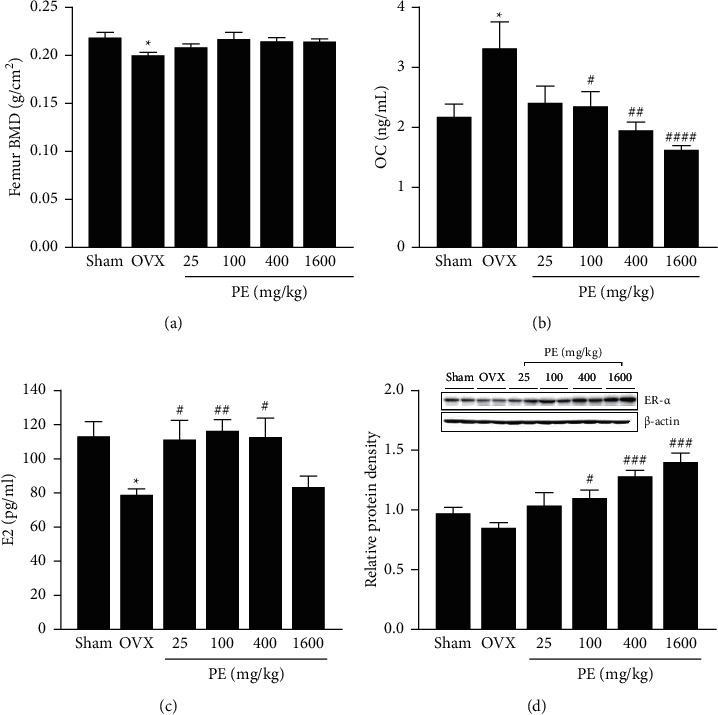
Effects of PE on (a) femur bone mineral density (BMD), (b) estradiol (E2), (c) osteocalcin (OC), and (d) estrogen receptor-alpha (ER-*α*) protein expression. Each column represents the mean ± standard error of mean (SEM). ^*∗*^*p* < 0.05*vs*. sham group; ^#^*p* < 0.05, ^##^*p* < 0.01, ^###^*p* < 0.001, and ^####^*p* < 0.0001*vs*. OVX group.

The bone mineral density (BMD) of the femur is shown in Figure 3(a). The BMD in the OVX group was decreased by 5% compared with that in the sham group. PE treatment did not affect ovariectomy-induced decrease in BMD. OC levels were markedly increased in the OVX group compared to the sham group (Figure 3(b)). However, PE treatment (100, 400, or 1600 mg/kg) significantly inhibited the elevated OC levels to values similar to those in the sham group. Plasma E2 levels were significantly lower in the OVX group than in the sham group. However, PE treatment, except for 1600 mg/kg, significantly increased E2 levels in OVX rats (Figure 3(c)). The expression of ER-*α* was decreased in OVX rats, and PE treatment upregulated ER-*α* expression in a dose-dependent manner (Figure 3(d)).

### 3.6. Effects of PE on Serotonin and Bone Turnover Markers

Table 3 summarises the changes in the levels of serotonin and bone turnover markers, such as CTX-1, DPD, and PYD. The OVX group showed a considerable increase in serotonin, DYP, and PYD levels compared with the sham group. There was no significant difference in CTX-1 levels between the sham and OVX groups. PE treatment of OVX rats significantly decreased these bone turnover markers and serotonin levels.

## 4. Discussion

Estrogen deficiency in postmenopausal women and OVX rats promotes metabolic syndromes [17]. The OVX rat model has been widely used to evaluate the estrogenic activity of natural compounds [18]. In this study, we investigated the estrogenic activity of PE against postmenopausal symptoms such as obesity, osteoporosis, and dyslipidemia in OVX rats.

The OVX rats present with weight gain, uterine atrophy, bone loss, and estrogen reduction, all of which commonly occur in postmenopausal women. As described previously [19], OVX rats showed a significant increase in body weight gain, distinct uterine atrophy, and uterine weight loss compared with sham-operated rats, indicating that ovariectomy successfully induced menopause. However, PE treatment inhibited uterine weight loss and body weight gain and increased endometrial thickness in OVX rats. These results suggest that PE could improve postmenopausal symptoms in OVX rats.

We also confirmed that PE supplementation elevated the expression of ER-*α* protein in the uterus and plasma E2 levels in OVX rats. ER-*α* is essential for the induction of uterine muscular disorganization and vaginal epithelial cell proliferation by estrogen during critical developmental stages [20]. These results imply that PE might exert estrogenic effects by increasing E2 levels and improving the uterus.

The supplementation with PE up to 1600 mg/kg did not change ALT and AST levels, which are biomarkers of hepatic injury. This result is similar to that of a previous study, which reported that *kudzu* roots are not highly toxic based on toxicity assessment [21]. These data suggest that PE intake at this dose did not cause any adverse effects on the livers of OVX rats.

Estrogen deprivation by ovariectomy leads to increased fat accumulation and hepatic steatosis [22]. The ER-*α* knockout female mice are reported to exhibit an obese phenotype with increased visceral adiposity, decreased energy expenditure, and insulin resistance [23, 24]. Similar to previous studies, the OVX group showed a significant increase in visceral fat mass, plasma FFA, insulin levels, and hepatic lipid peroxidation. The ER-*α* is also necessary for the attenuation of body weight gain after OVX [25]. In this study, PE treatment significantly suppressed body weight gain, abdominal fat accumulation, and blood lipid levels. These results suggest that PE acts as a phytoestrogen in lipid metabolism in OVX rats.

Blood Ca and P levels reflect the degree of bone loss [26]. This study showed that Ca levels in the OVX group were slightly lower than those in the sham group. However, PE treatment (400 and 1600 mg/kg) significantly increased the blood Ca levels in OVX rats. Biochemical markers of bone turnover have been widely used to assess the metabolic activity of bone remodeling [27, 28]. The results indicated that femur BMD was lower in OVX rats than in sham rats. Bone turnover was increased by OVX-induced estrogen deficiency, as evidenced by higher bone resorption (CTX, DPD, and PYD) and higher bone formation (OC) in OVX rats than in sham rats. These findings are consistent with those of previous studies [29, 30]. In contrast, PE supplementation significantly suppressed the increase in bone turnover marker levels, suggesting that PE may have sufficient estrogenic activity to affect the bone turnover rate in OVX rats.

It has been reported that blood serotonin levels increase with an increase in bone turnover in humans and in an OVX rat model of postmenopausal osteoporosis [31]. In the present study, we found that circulating serotonin significantly increased in the OVX group. However, PE treatment significantly inhibited the increase in serotonin levels. Therefore, serotonin levels may be used as a marker of postmenopausal osteoporosis.

## 5. Conclusions

In the present study, it was demonstrated that PE exhibits phytoestrogen-like activities in OVX rats. PE supplementation increased the plasma estradiol level, uterine layer thickness, and ER-*α* protein expression, without affecting serum levels of hepatic injury biomarkers. Moreover, PE treatment inhibited the increase in bone turnover markers such as CTX-1, OC, DYP, and PYD in OVX rats. Overall, PE might be a potential functional food for controlling menopausal symptoms.

## Figures and Tables

**Table 1:** Effects of PE on plasma biochemical parameters in OVX rats.

	Sham	OVX	PE-25	PE-100	PE-400	PE-1600
ALT (U/L)	17.46 ± 1.27	24.70 ± 2.51	24.87 ± 1.99	23.76 ± 1.31	20.77 ± 0.98	22.01 ± 1.60
AST (U/L)	76.48 ± 5.11	109.00 ± 16.84	99.76 ± 6.44	93.73 ± 6.51	80.73 ± 6.37	74.90 ± 5.29
Ca (mg/dL)	4.95 ± 0.31	4.47 ± 0.40	5.28 ± 0.12	5.28 ± 0.14	5.73 ± 0.13^##^	5.72 ± 0.12^##^
P (mg/dL)	4.06 ± 0.22	4.49 ± 0.56	4.37 ± 0.30	4.28 ± 0.32	4.91 ± 0.20	4.45 ± 0.25
FFA (*μ*M)	197.3 ± 14.96	346.7 ± 35.42^*∗*^	254.2 ± 33.97	247.7 ± 37.05	321.40 ± 37.74	173.7 ± 42.8^##^
Insulin (ng/mL)	0.92 ± 0.04	1.23 ± 0.03^*∗∗∗*^	1.00 ± 0.04^##^	1.11 ± 0.05	1.03 ± 0.05^##^	0.94 ± 0.04^###^

Data are presented as the mean ± standard error of mean (SEM). Sham, sham operation; OVX, ovariectomy; PE-25, OVX + PE 25 mg/kg; PE-100, OVX + PE 100 mg/kg; PE-400, OVX + PE 400 mg/kg; PE-1600, OVX + PE 1600 mg/kg; FFA, free fatty acid; ALT, alanine aminotransferase; AST, aspartate aminotransferase. ^*∗*^*p* < 0.05 and ^*∗∗∗*^*p* < 0.001 vs. OVX group; ^##^*p* < 0.01 and ^###^*p* < 0.001*vs*. OVX group.

**Table 2:** Effects of PE on plasma lipid profiles and liver MDA levels in OVX rats.

	Sham	OVX	PE-25	PE-100	PE-400	PE-1600
TC (mg/dL)	48.25 ± 3.09	60.10 ± 4.34	55.45 ± 5.62	65.64 ± 1.88	61.81 ± 2.92	55.52 ± 3.78
HDL-C (mg/dL)	55.53 ± 2.68	59.30 ± 4.85	72.52 ± 5.01	71.20 ± 3.15	74.46 ± 4.18^#^	65.60 ± 3.16
LDL-C (mg/dL)	7.76 ± 0.38	10.96 ± 0.82	10.83 ± 1.14	11.23 ± 0.51	9.80 ± 0.79	9.27 ± 0.91
TG (mg/dL)	38.71 ± 3.71	59.55 ± 3.96^*∗∗*^	33.74 ± 5.49^###^	44.13 ± 2.27^#^	42.84 ± 2.01^#^	49.78 ± 4.03
MDA (*μ*M)	47.95 ± 2.46	60.22 ± 3.39^*∗*^	42.05 ± 2.64^###^	39.9 ± 2.23^####^	40.21 ± 2.11^####^	42.02 ± 4.02^###^

Data are presented as the mean ± standard error of mean (SEM). Sham, sham operation; OVX, ovariectomy; PE-25, OVX + PE 25 mg/kg; PE-100, OVX + PE 100 mg/kg; PE-400, OVX + PE 400 mg/kg; PE-1600, OVX + PE 1600 mg/kg; TC, total cholesterol; HDL-C, high-density lipoprotein cholesterol; LDL-C, low-density lipoprotein cholesterol; triglyceride, TG; MDA, malondialdehyde. ^*∗*^*p* < 0.05 and ^*∗∗*^*p* < 0.01*vs*. sham group; ^#^*p* < 0.05, ^###^*p* < 0.001, and ^####^*p* < 0.0001*vs*. OVX group.

**Table 3:** Effects of PE on serotonin and bone turnover markers in OVX rats.

	Serotonin (ng/mL)	CTX-1 (pg/mL)	DYP (nmol/L)	PYD (ng/mL)
Sham	200.10 ± 28.19	167.10 ± 14.10	2.10 ± 0.10	27.07 ± 0.97
OVX	390.20 ± 67.26^*∗*^	155.5 ± 8.14	3.07 ± 0.18^*∗∗∗*^	34.92 ± 3.20^*∗*^
PE-25	264.30 ± 38.04	106.80 ± 7.95^##^	2.37 ± 0.07^###^	27.47 ± 1.51^#^
PE-100	205.70 ± 22.92^#^	110.60 ± 7.14^#^	2.23 ± 0.12^####^	25.86 ± 1.42^##^
PE-400	222.60 ± 40.00^#^	104.10 ± 11.25^##^	2.34 ± 0.08^###^	25.18 ± 1.06^###^
PE-1600	159.40 ± 45.05^##^	96.24 ± 9.87^###^	2.09 ± 0.10^####^	25.31 ± 0.88^##^

Data are presented as the mean ± standard error of mean (SEM). Sham, sham operation; OVX, ovariectomy; PE-25, OVX + PE 25 mg/kg; PE-100, OVX + PE 100 mg/kg; PE-400, OVX + PE 400 mg/kg; PE-1600, OVX + PE 1600 mg/kg; CTX-1, C-terminal telopeptide fragment of type I collagen; DYP, deoxypyridinoline; PYD, pyridinoline. ^*∗*^*p* < 0.05 and ^*∗∗∗*^*p* < 0.001*vs*. sham group; ^#^*p* < 0.05, ^##^*p* < 0.01, ^###^*p* < 0.001, and ^###^*p* < 0.0001*vs*. OVX group.

## Data Availability

The data used to support the findings of this study are included within the article.
